# Changes of Peripheral Th17 Cells Subset in Overweight and Obese Children After Body Weight Reduction

**DOI:** 10.3389/fendo.2022.917402

**Published:** 2022-07-06

**Authors:** Dorota Artemniak-Wojtowicz, Anna M. Kucharska, Anna Stelmaszczyk-Emmel, Anna Majcher, Beata Pyrżak

**Affiliations:** ^1^Department of Pediatrics and Endocrinology, Medical University of Warsaw, Warsaw, Poland; ^2^Department of Laboratory Diagnostics and Clinical Immunology of Developmental Age, Medical University of Warsaw, Warsaw, Poland

**Keywords:** children, obesity, metabolic complication, Th17 cells, inflammation

## Abstract

**Background:**

Obesity has been a growing problem in young patients leading to serious metabolic complications. There are many studies supporting the idea, that obesity should be considered as a chronic inflammation closely associated with immune system alterations. Th17 subpopulation is strongly involved in this process. The aim of our study was to evaluate circulating Th17 cells in overweight and obese children and explore the relationships between Th17 subset and metabolic parameters.

**Methods:**

We evaluated peripheral Th17 cells in fresh peripheral blood samples from 27 overweight and obese and 15 normal-weight children. Th17 cells were identified by flow cytometry using monoclonal antibody and intracellular IL-17A staining. Th17 cells were defined as CD3^+^CD4^+^CD196^+^IL-17A^ic+^. The analysis involved anthropometric and metabolic parameters measured at baseline and three months after the change of lifestyle and diet. We evaluated the relationship between metabolic parameters and Th17 cells.

**Results:**

In overweight and obese children we found significantly higher Th17 cells percentage compared to normal weight controls (median 0.097% (0.044 - 0.289) vs 0.041% (0.023 - 0.099), p = 0.048). The percentage of Th17 cells decreased statistically significantly in children who reduced weight after the intervention (0.210% (0.143 - 0.315) vs 0.039% (0.028 - 0.106), p = 0.004). In this group we also noticed statistically significant reduction of TC and LDL-C concentration (p = 0.01, p = 0.04, respectively).

**Conclusions:**

Obesity in children is associated with increased percentage of peripheral Th17 cells. Weight reduction leads to significant decrease of circulating Th17 cells and improvement of lipid parameters. This significant reduction of proinflammatory Th17 cells is a promising finding suggesting that obesity-induced inflammation in children could be relatively easily reversible.

## Introduction

In recent decades obesity has become a global epidemic (1). This is also an increasing problem in younger and younger patients (2), leading to pathological changes. In the light of recent data, adipose tissue (AT) has been considered as an active organ involved in numerous immunological, hormonal and metabolic processes (3). Obesity should be recognized as a factor contributing to the development of generalized low-grade sterile chronic inflammation, closely associated with morphological and functional changes in AT. These alterations include the colonization of AT by increased number of macrophages with its phenotypic switch from M2 to M1, infiltration of mast cells and neutrophils, imbalance between Th17 cells and Treg (4, 5) as well as tissue hypoxia and oxidative stress (6). Just as long-term obesity leads to fixation of structural changes in adipose tissue, as obesity-induced chronic inflammation also may cause difficult to reverse metabolic implications (3), however the mechanisms of this phenomena are still not fully explained. Many studies presented the role of obesity-induced inflammation in the development of such conditions as insulin resistance (IR), type 2 diabetes (T2DM), bronchial asthma, inflammatory bowel disease, rheumatoid arthritis, psoriasis and certain types of cancer (7–9).

Since 2005, when the Th17 lymphocytes subpopulation was described for the first time, it has been attributed to the inflammatory processes (10, 11). Their contribution in the antibacterial and antifungal immunity (7, 12) as well as their influence on the development of autoimmune and allergic disorders has been well documented (13, 14).

Th17 cells are characterized by expression of retinoid acid receptor (RAR)-related orphan receptor C (RORC) – master transcription factor. RORC is essential for the differentiation of naive Th cells into Th17 subset and important in producing Th17 cells’ cytokines, whose signature product is interleukin (IL)-17A and IL-17F (15–17).

The Th17 lymphocytes could be the important link between obesity and inflammation (8). An increased number of Th17 cells in the spleen and AT was found in animal models of obesity (8, 18). Likewise, increased Th17 cells frequency was found in peripheral blood in humans with obesity and diabetes mellitus (19).

The aim of our study was to evaluate alterations of peripheral Th17 cells subset in overweight and obese children before and after weight reduction and to explore its potential metabolic implications.

## Materials and Methods

The study was performed in the Department of Pediatrics and Endocrinology of Medical University of Warsaw, between August 2016 and October 2020, and was approved by the Bioethics Committee at the Medical University of Warsaw. Written informed consent was obtained from parents and simultaneously from patients older than 16 years.

27 children: 7 overweight and 20 obese, between 8 to 18 years of age (mean age 12.76 years, range 8.33 - 17.58) were enrolled to the study: 12 girls and 15 boys. The control group consisted of 15 normal weight children age-matched (7 girls and 8 boys). The study group of children had a median body mass index standard deviation score (BMI SDS) of 2.3 (1.9 - 2.6) in comparison to 0.1 (-0.5 - 0.3) in the control group (p < 0.005). Almost in 89% of overweight and obese children, their WHtR met the criteria of abdominal obesity. The patient’s anthropometric data are shown in **Table 1**.

**Table 1:** Characteristic of anthropometric measurements in overweight and obese children (study group) and normal weight children (control group).

Variable	Study group (n = 27)	Control group (n = 15)	*p-*value
BMI (kg/m^2^)	30.2 (27.2 - 34)	18.6 (16.5 - 20.2)	<0.001
BMI SDS	2.3 (1.9 - 2.6)	0.1 (-0.5 - 0.3)	<0.001
WC (cm)	94.4 ± 13.94	63.91 ± 3.36	<0.001
WC SDS	3.7 (2.6 - 4.4)	-0.3 (-0.7 - 0.1)	<0.001
HC (cm)	106.00 (95.00 - 109.00)	80.00 (74.00 - 87.00)	<0.001
WHR	0.88 ± 0.055	0.80 ± 0.05	<0.001
WHtR	0.56 (0.53 - 0.6)	0.42 (0.40 - 0.44)	<0.001

Data are presented as median values with interquartile range as appropriate or mean ± standard deviation (SD); BMI - body mass index, BMI SDS - body mass index standard deviation score, WC - waist circumference, HC - hip circumference, WHR - waist-to-hip ratio, WHtR - waist-to-height ratio. A p < 0.05 was considered significant.

Obesity was defined by BMI > +2 SDS based on a nationally representative group of children aged 3-18 years (20). Overweight was defined by BMI between +1 to +1.9 SDS. Secondary obesity due to central nervous system diseases, hormonal or genetic disorders was the criterion of exclusion.

The analysis involved parameters measured at baseline and three months after the change of lifestyle and diet. The baseline visit included a dietary intervention performed by a dietician, who recommended: 5 meals a day at intervals of 2.5-3 h, no sweet drinks, reduction of simple sugars, meals with a low glycemic index. The energy supply was appropriate for sex, age and physical activity. Additionally, a minimum of 60 minutes of physical activity was recommended daily.

A control group consisted of children with normal somatic parameters (BMI < +1SDS) who were age- and sex-matched.

All blood samples were obtained by peripheral venipuncture in the morning after an overnight fasting. In the study group: at baseline and after three months of intervention and in the control group only at baseline.

In all children the allergy, hematological or chronic disease and symptoms of acute infection were excluded by medical history and physical examination.

### Laboratory Methods

#### Flow Cytometry

50µl of fresh whole blood was stained with 5 µl of monoclonal antibodies (according to manufacturer’s instructions, Becton Dickinson Biosciences): anti-CD3 APC-H7; anti-CD4 PE-Cy7; anti-CD196 APC (CCR6) (Becton Dickinson, Franklin Lakes, NJ, USA). The samples were incubated for 20 minutes in the dark at room temperature. Next, the cells were incubated 15 minutes in 100µl of permeabilization buffer- IntraPrep Permabilization Reagent 1(Immunotech SAS, Beckman Coulter Company, 13276 Marseille Cedex 9, France) in room temperature in the dark. Then, cells were washed in a washing buffer (0.9% NaCl) for 5 minutes, 500 g. Afterwards, cells were incubated 5 minutes in room temperature in the dark with 100µl of permeabilization buffer-IntraPrep Permabilization Reagent 2 (Immunotech SAS, Beckman Coulter Company, 13276 Marseille Cedex 9, France), next washed in washing buffer, centrifuged at 500g for 5 minutes. Subsequently, the cells were stained with 20µl anti-IL-17A PE monoclonal antibody for 20 minutes in room temperature in the dark and washed. Cells were stored in room temperature before analysis. For staining procedures appropriate isotype-matched controls were used.

Flow cytometry was performed on FACS Canto II flow cytometer (Becton Dickinson, Franklin Lakes, NJ, USA) using BD FACS Diva 8.0.1 software. Gates were preset and the measurements were performed blinded for sample identity. Th17 cells were defined as CD3^+^CD4^+^CD196^+^ expressing intracellularly interleukin-17A (CD3^+^CD4^+^CD196^+^IL-17A^ic+^). The number of Th 17 cells was expressed as a percentage values.

**Figure 1:**
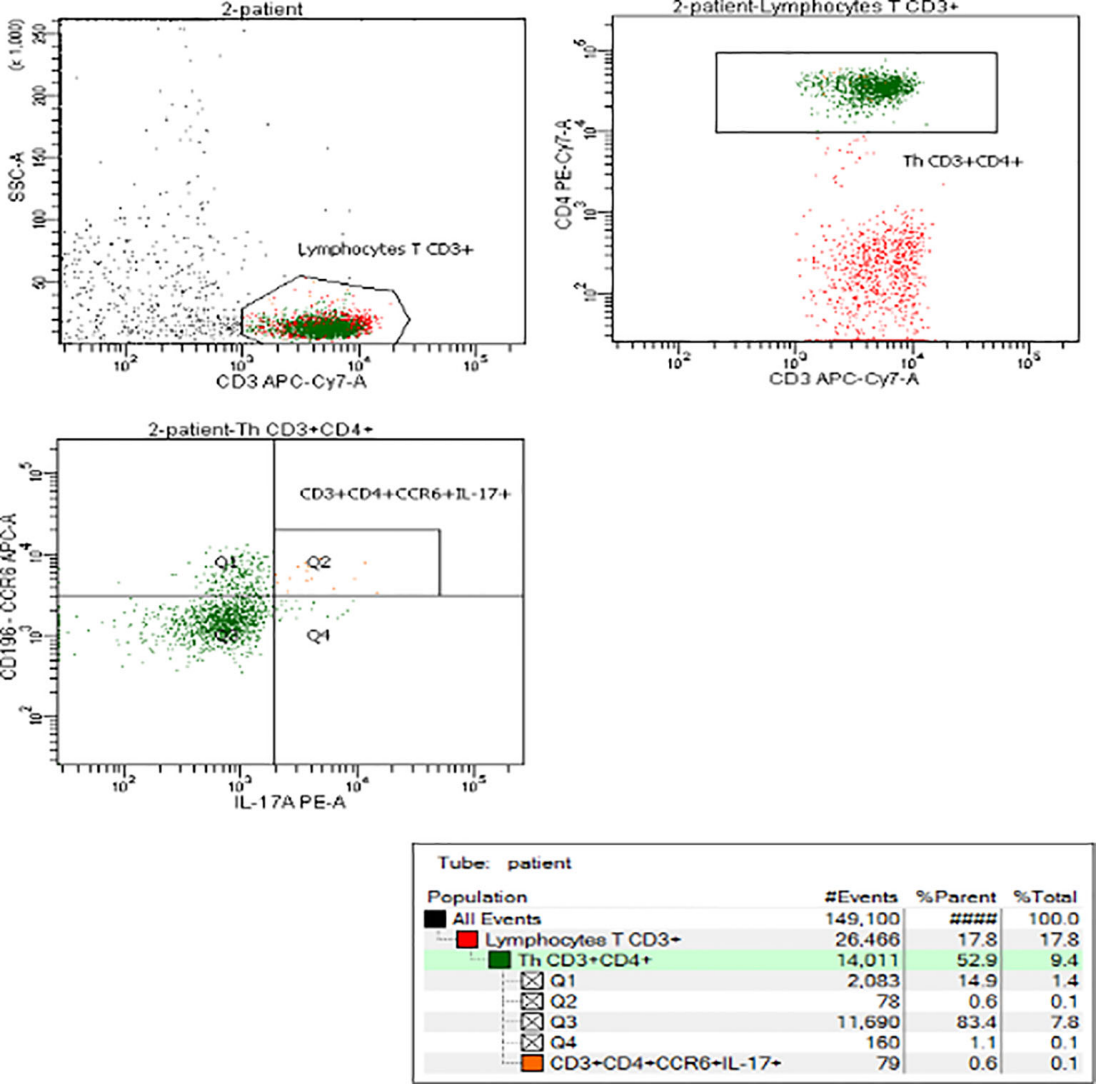
Representative flow cytometry analysis of Th17 cells in obese child.

Representative flow cytometry analyses of Th17 cells, with gating algorithms, are shown in the **Figure 1**.

#### Biochemical Parameters

Blood samples were analyzed by standard methods.

Concentration of C-reactive protein (CRP) (mg/dl) was determined using a fix-point immune-rate method, on the Vitros 5600 analyzer (Ortho Clinical Diagnostic, Raritan, New Jersey, USA).

In obese and overweight’s children the Oral Glucose Tolerance Test (OGTT) (1.75 g of glucose/kg body weight, no more than 75 g) with insulin level evaluation was performed after an overnight fasting. Blood samples were taken at 0-, 30-, 60-, 90- and 120-min. Glycated hemoglobin (HbA1c) was also measured. Blood tests in control group included fasting glucose, insulin concentration and HbA1c. The concentration glucose was measured by glucose oxidase colorimetric method using Vitros 5600 analyzer (Ortho Clinical Diagnostic, Raritan, New Jersey, USA). Insulin values were measured in serum by immunoassay using IMMULITE 2000 Xpi Analyzer (Siemens, Erlangen, Germany). HbA1c was measured in whole blood by ion-exchange high-performance liquid chromatography (HPLC) using D-10 Hemoglobin Analyzer (BIO-RAD, California, USA). The values of fasting glucose, insulin and HbA1c were compared to the normal ranges recommended for healthy children. Fasting insulin level ≥ 15 μIU/ml was considered elevated (21). Homeostasis model assessment insulin resistance index (HOMA-IR) and the quantitative insulin sensitivity check index (QUICKI) were calculated based on concentrations of fasting glucose (mg/dl) and fasting insulin (μIU/ml) (22) and have been established as an indicator of IR. The HOMA-IR was calculated as follows: HOMA-IR= [glucose (mmol/l) x insulin (μIU/ml)]/22.5, glucose conversion factor: mmol/l=mg/dl x 0.05551. The QUICKI was calculated as follows: QUICKI=1/[log insulin (μIU/ml) + log glucose (mg/dl). Glucose metabolism and lipid profile parameters were evaluated based on the Polish Diabetes Association’ (PTD, 2021) (23) and the Polish Society of Laboratory Diagnostics (PSLD,2020) and the Polish Lipid Association (PoLA,2020) recommendations (24).

Lipid profile: total cholesterol (TC mg/dl), triglycerides (TG, mg/dl), and high-density lipoprotein cholesterol (HDL-C, mg/dl) concentrations were measured by the colorimetric enzymatic method using the Vitros 5600 analyzer (Ortho Clinical Diagnostic, Raritan, New Jersey, USA). Low-density lipoprotein cholesterol (LDL-C) was calculated using Friedewald formula [LDL-C= TC-(HDL+TG/5)]. The TG: HDL-C ratio were calculated; the value ≥ 3 (25) were consisted as closely correlated with IR.

Aspartate and alanine aminotransferase activity (AST and ALT) was measured by dry chemistry method using Vitros 5600 analyzer (Ortho Clinical Diagnostic, Raritan, New Jersey, USA).

### Anthropometry

Body weight (kg) was measured by means of medical scales to the nearest 0,1 kg, height (cm)- using a stadiometer (Holtain Limited) to the nearest 0,1 cm. Waist and hip circumferences were measured by a flexible measuring tape, according to WHO recommendations (26). Waist circumference (cm) was measured midway between the 10^th^ rib and the top of the iliac crest. Height, weight and waist circumferences were normalized for calendar age according to polish national references- the OLAF project (20, 27). Based on these measurements the waist-to-hip ratio (WHR) and waist-to-height ratio (WHtR) were calculated. WHtR exceeding 0.5 was assumed to define abdominal obesity (28). BMI was calculated: weight (kg) divided by height in square meters (m^2^); BMI has been standardized with OLAF norms using the least mean square method (LMS) and converted as BMI in SDS. Anthropometric measurements were taken by one anthropologist, patient was wearing only underwear, standing in the anthropometric position.

### Data Analysis

Detailed statistical calculations were performed using SPPS 13.3 software. Shapiro-Wilk test was used to check the normality of distribution. Data were presented as means and standard deviation (SD) for normally distributed data or median with interquartile range (IQR), when the distribution was different from normal. In comparison between overweight and obese children and control group a Student’s T-test (parametric data) or U Mann*-*Whitney test (nonparametric data) was performed, as appropriate. Relationship analysis were performed using the Spearman’s rank correlation coefficient test. A p-value < 0.05 was considered as statistically significant.

## Results

### Flow Cytometry

#### At Baseline

##### Increased Th17 Cell Frequency in Obese and Overweight Children

**Figure 2:**
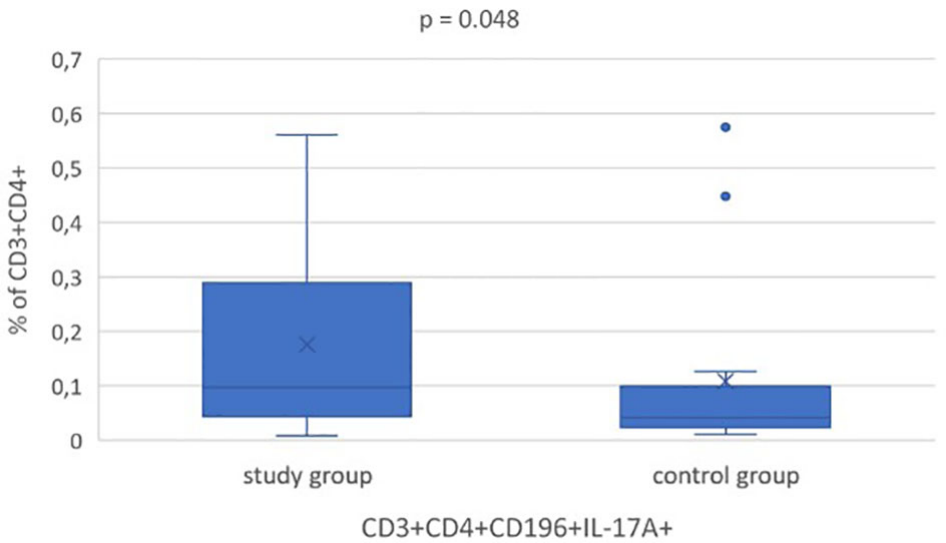
Statistical analysis of Th17 (CD3^+^CD4^+^CD196^+^IL-17A^+ic^) cells frequency at baseline in overweight and obese children (study group) and normal weight ones (control group). All box plots represent the minimum, the first quartile, the median, the mean, the third quartile and the maximum. Dots represent outliers.

Initially, there was analyzed the subgroup of obese in comparison to overweight children and no statistical difference in Th17 cells frequency was found. In the study group there was statistically significant higher frequency of Th17 cells in the peripheral blood in comparison to normal-weight controls; median value 0.097% *vs* 0.041%, respectively (p = 0.048; **Figure 2**). In the group of all children, the percentage of Th17 lymphocytes correlated positively with BMI (p = 0.048, r = 0.31), BMI SDS (p = 0.032, r = 0.33) and WHtR (p = 0.02, r = 0.37). In overweight and obese group there was statistically significant correlation between Th17 frequency and WHR (p = 0.005, r = 0.54).

### Follow-Up Visit

#### The Changes of Th17 Cells Percentage After Weight Loss

After three months, 21 overweight and obese children (10 girls and 11 boys) were assessed; 6 children didn’t attend the visit. 12 of children lost weight (ΔBMI SDS <0 (-0.4 ≥ ΔBMI SDS ≥ -0.1)), 5 gained weight (ΔBMI SDS >0) and in 4 ones BMI SDS (ΔBMI SDS =0) remained unchanged.

**Figure 3:**
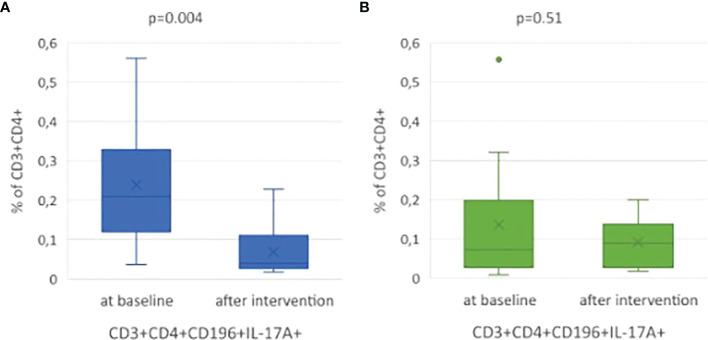
**(A)** Statistical analysis amount of Th17 cells (%) in the study subgroup at baseline and three months later after BMI reduction. **(B)** Statistical analysis of Th17 cells frequency in the study subgroup at baseline and after intervention, without BMI reduction. All box plots represent the minimum, the first quartile, the median, the mean, the third quartile and the maximum. Dots represent outliers.

In the group of children who reduced weight (ΔBMI SDS <0, p <0.005) after the intervention, the percentage of Th17 cells decreased statistically significantly: 0.210% vs 0.039%, p = 0.004 (**Figure 3A**) and reached the value similar to the control group (0.041%).

In 9 children, who did not decrease BMI SDS, we did not observe any statistically significant changes in Th17 frequency (p = 0.51; **Figure 3B**).

### Biochemical and Metabolic Parameters

#### At Baseline

##### Inflammation

CRP concentration was similar in overweight and obese children (0.5 (0.5-0.5)) and controls (0.5 (0.5-0.5)), p = 0.78. In overweight and obese children, we reported a positive correlation between CRP concentration and BMI SDS (p = 0.01, r = 0.47), WC SDS (p = 0.009, r = 0.49) and WHtR (p = 0.001, r = 0.59). In all children, a positive correlation was confirmed between CRP concentration and BMI SDS (p = 0.043, r = 0.3), but there was no correlation between CRP concentration and the percentage of Th17 cells in peripheral blood.

##### Glucose Metabolism

**Figure 4:**
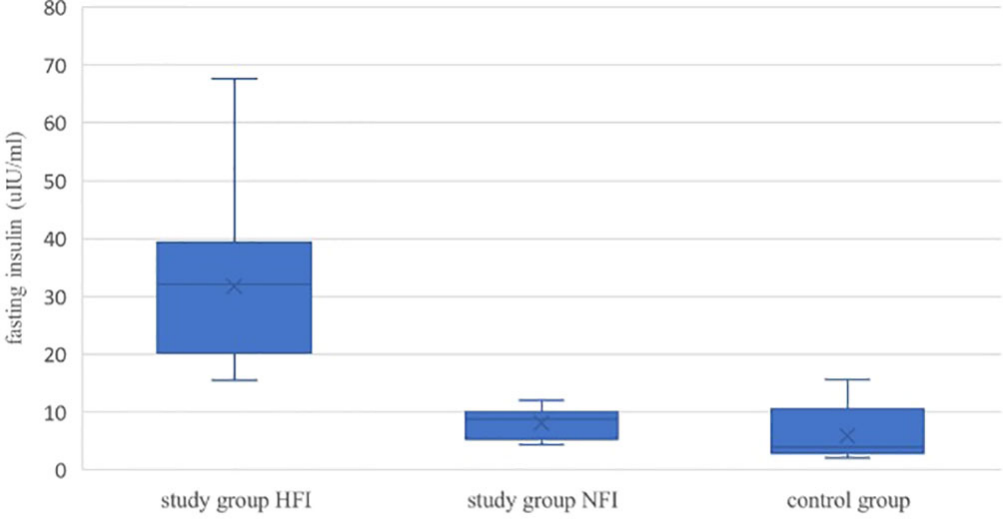
Comparison of fasting insulin concentration in overweight/obese children and controls. Study group HFI v study group NFI p < 0.001, study group HFI v control group p < 0.001, study group NFI v control group p = 0.11. HFI- high fasting insulin, NFI- normal fasting insulin. All box plots represent the minimum, the first quartile, the median, the mean, the third quartile and the maximum. A p < 0.05 was considered significant.

All children had normal fasting glucose concentration and HbA1c percentage, but among overweight and obese children 19 out of 27 (70%) had elevated fasting insulin concentration (**Figure 4**). In all children it was found a statistically significant correlation between Th17 cells percentage and the parameters of carbohydrate metabolism: fasting insulin (p = 0.01, r = 0.39), HOMA-IR (p = 0.01, r = 0.38), QUICKI (p = 0.015, r = -0.37). In obese and overweight children, the statistically significant positive correlation was found between the frequency of Th17 lymphocytes and the concentration of glucose and insulin 2 h after OGTT (p = 0.017, r = 0.45; p = 0.006, r = 0.52, respectively).

##### Lipids

14 out of 27 overweight/obese children (52%) had an increased TC concentration, 9 children (33%) LDL-C, 20 ones (74%) TG concentration; 15 children (55.5%) had decreased HDL-C concentration. In all children we found a statistically significant correlation between Th17 cells percentage and concentration of HDL-C (p = 0.046, r = -0.31) and TG (p = 0.027, r = 0.35), as well as TG: HDL-C ratio (p = 0.015; r = 0.38).

##### Liver

At baseline in overweight and obese children we found increased ALT and AST activity in 41% (11/27) and 22% (7/27), respectively. Aminotransferases’ activity correlated positively with CRP concentration (ALT p = 0.018, r = 0.46; AST p = 0.006, r = 0.51). Moreover, we detected positive correlation between AST activity and CRP concentration in normal weight ones (p = 0.04; r = 0.57). In all children we noticed a tendency to correlation between Th17 lymphocytes and ALT activity but without statistical significance (p = 0.07, r = 0.29). The comparison of biochemical parameters at baseline in overweight and obese to normal weight children are reported in **Table 2**.

**Table 2:** Biochemical parameters in overweight and obese children in comparison to normal weight children.

Variable	Obese children (n = 27)	Normal weight children (n = 15)	p-value
CRP (mg/dl)	0.5 (0.5 - 0.5)	0.5 (0.5 - 0.5)	ns
ALT (U/L)	35 (27 - 47)	18.00 (16.0 - 22.0)	<0.001
AST (U/L)	31 (27 - 38)	30.00 (22.0 - 33.0)	ns
Fasting glucose (mg/dl)	85.96 ± 7.22	83 ± 5.7	ns
Fasting insulin (μIU/ml)	20.4 (10.1 - 36.7)	3.92 (2.89 - 10.6)	<0.001
HbA_1_c (%)	5.3 (5.15 - 5.45)	5.3 (5.0 - 5.4)	ns
HOMA-IR	3.8 (2.09 - 7.66)	0.79 (0.58 - 2.2)	<0.001
QUICKI	0.31 ± 0.03	0.39 ± 0.05	<0.001
TC (mg/dl)	161.4 ± 34.76	178.14 ± 24.58	ns
HDL-C (mg/dl)	41.04 ± 9.45	63.28 ± 11.35	<0.001
LDL-C (mg/dl)	93.2 (71.4 - 115.6)	95.9 (81.2 - 121.0)	ns
TG (mg/dl)	145.93 ± 63.5	78.0 ± 20.37	<0.001

Data are presented as mean ± standard deviation (SD) or median values with interquartile range as appropriate; CRP, C-reactive protein; ALT, alanine aminotransferase activity; AST, aspartate aminotransferase activity; HbA1c, glycated haemoglobin; HOMA-IR, homeostasis model assessment-insulin resistance; QUICKI, quantitative insulin sensitivity check index; TC, total cholesterol; HDL-C, high-density lipoprotein cholesterol; LDL-C, low-density lipoprotein cholesterol; TG, triglycerides; ns, non-significant. A p < 0.05 was considered significant.

Additionally, we compared the biochemical parameters in subgroup of overweight and obese children, and we found statistically significant differences only in the value of ALT, HDL-C, fasting insulin and HOMA-IR (**Table 3**).

**Table 3:** Biochemical differences between overweight and obese children.

Variable	Overweight children (n = 7)	Obese children (n = 20)	p-value
ALT	27 (24 - 30)	41 (31 - 71)	0.02
HDL-C	47.1 ± 6.8	38.9 ± 9.4	0.04
Fasting insulin	15.5 (6.25 - 20.2)	30.05 (13.75 - 38.8)	0.03
HOMA-IR	3.25 (1.4 - 3.8)	6.08 (3.03 - 8.13)	0.048

Data are presented as mean ± standard deviation (SD) or median values with interquartile range as appropriate; ALT, alanine aminotransferase activity; HDL-C, high-density lipoprotein cholesterol; HOMA-IR, homeostasis model assessment-insulin resistance. A p < 0.05 was considered significant.

### Follow-Up Visit

As reported in **Table 4**, in overweight/obese children, who reduced BMI after intervention, only TC (p = 0.01) and LDL-C (p = 0.04) improved significantly. There was also decreased insulin level but without statistical significance (p = 0.27).

**Table 4:** Comparison of biochemical parameters in subgroup of overweight/obese children at baseline and after BMI reduction (n=12).

Variable	At baseline	After BMI reduction	p-value
CRP (mg/dl)	0.5 (0.5 - 0.5)	0.5 (0.5 - 0.5)	0.65
ALT (U/l)	35 (30 - 43.5)	33 (27 - 39)	0.2
AST (U/l)	31.5 (28 - 35.5)	29.5 (26 - 34.5)	0.13
Fasting glucose (mg/dl)	84 ± 7	84 ± 4.6	0.8
Fasting insulin (μIU/ml)	30.1 (20.3 - 36.1)	21.5 (14.4 - 35.3)	0.27
HbA1c (%)	5.35 (5.2 - 5.45)	5.15 (4.8 - 5.5)	0.33
HOMA-IR	6.08 (3.71 - 7.62)	4.6 (2.9 - 7.5)	0.31
QUICKI	0.295 (0.29 - 0.315)	0.305 (0.29 - 0.325)	0.17
TC (mg/dl)	161.6 ± 27.5	148 ± 27	0.01*
HDL-C (mg/dl)	37.8 ± 9.3	38.7 ± 11.4	0.61
LDL-C (mg/dl)	90.9 ± 18	83.5 ± 20	0.04*
TG (mg/dl)	162 (113 - 198)	108 (89 - 169)	0.08
TG/HDL-C ratio	3.78 (2.85 - 5.45)	2.66	0.16

Data are presented as mean ± standard deviation (SD) or median values with interquartile range as appropriate; CRP, C-reactive protein; ALT, alanine aminotransferase activity; AST, aspartate aminotransferase activity; HbA1c, glycated haemoglobin; HOMA-IR, homeostasis model assessment-insulin resistance; QUICKI, quantitative insulin sensitivity check index; TC, total cholesterol; HDL-C, high-density lipoprotein cholesterol; LDL-C, low-density lipoprotein cholesterol; TG, triglycerides; TC/HDL-C ratio. A p < 0.05* was considered significant.

In the group of children without BMI reduction after intervention, no significant changes in biochemical parameters as well as insulin level were observed.

## Discussion

Th17 cells, a subset of CD4^+^ helper T cells, like other immune cells, are recognized by expression of characteristic patterns of master transcription factors, cytokine production profiles and extracellular proteins. In the studies using flow cytometry, there are applied many different staining protocols of Th17 identification (19, 29, 30). In our study, the subset of Th17 cells from human’s peripheral blood was defined by the expression of CD196 (CCR6) (31, 32) on the CD3^+^ CD4^+^ cell surface and intracellular interleukin (IL)-17A presence (CD3^+^CD4^+^CD196^+^IL-17A^ic+)^.

Th17 cells are a small population of cells and their stimulation, including a.o. ionomycin/PMA, is typically used for their evaluation *in vitro*. The use of these stimulants is a common method for assessing Th17 lymphocytes activity in flow cytometry but requires incubation for several hours. Therefore, we performed an easier and shortened protocol with reduced cell manipulation *in vitro* that can be used simultaneously in a clinical setting. Our study evaluated the spontaneous intracellular expression of IL-17A in peripheral Th17 cells without stimulation, and yet we obtained a statistically significant difference in the frequency of CD3^+^CD4^+^CD196^+^IL-17A^ic+^ cells in overweight/obese and normal weight children.

There is growing evidence, that the percentage of Th17 lymphocytes increases in obesity (32). Nevertheless, the mechanism initiating the Th17 immune response in humans with obesity is not fully understood. It seems that dendritic cells with CD11c^+^CD1c^+^ phenotype, present in the AT in mouse and humans, may play an important role in this process (33).

Interestingly, in animals it was found a higher level of CD4^+^IL-17^+^T cells in AT of high fat diet (HFD) mice compared to the lean ones (18). Likewise, higher amount Th17 cells were detected in the spleens of HFD mice compared to normal mice (8). The contribution of Th17 cells in AT was also assessed in human (34) and in obese individuals Th17 cells were markedly increased in visceral AT, moreover their number strongly correlated with peripheral blood Th17 subset (35). However, for clinical use, the studies based on the parameters of peripheral blood seem to be more valuable because of its availability. In our opinion, research using less invasive and relatively easily reproducible methods could be more useful, especially in children.

In our study, the statistical significance of the difference of the Th17 frequency was at the borderline (p = 0.048). Nevertheless, our results are in line with data of Schindler (36), who in their study observed that the frequency of circulating Th17 cells was significantly increased in overweight children compared to non-overweight controls in the absence of acute or chronic inflammatory diseases. Furthermore, they found a significantly higher expression of RORC- and IL-17A-mRNA transcripts after stimulation in PBMCs from overweight children (36). In the study of Łuczyński et al. it was detected that both children with central obesity and children with long-term diabetes type 1 (DM1) have elevated levels of CD4^+^CD161^+^CD196^+^IL-17^+^ cells in the peripheral blood (19).

Calcaterra et al. (37) evaluating the Th17 and Treg lymphocyte balance in obese and normal weight children reported different results. They observed a decreasing trend of circulating Th17 cells in children with obesity compared with normal weight ones, without statistical significance (37). Nevertheless, after dividing these patients into two subgroups: metabolically healthy (MH) or unhealthy (MU), a higher percentage of Th17 cells was observed in the MU group, but this result was not statistically significant (37). However, most studies confirm higher levels of Th17 cells in obese patients (19, 38, 39). Furthermore, the results of our study, as well as Schindler’s data (36), detected a positive correlation between the frequency of Th17 cells and BMI. Additionally, we reported for the first time, to our knowledge, that after BMI reduction in obese children Th17 cells frequency statistically significant decreased. We also observed a positive correlation between Th17 cells and WHR or WHtR, which is considered as a sensitive marker of visceral adiposity (40).

The obesity-induced low-grade chronic inflammation is considered to predispose to metabolic disorders. The link between systemic sterile inflammation and increased Th17 cells and development of IR and T2DM was documented in several studies (34, 38, 41, 42). Fabrini et al. (43) showed that the AT from obese insulin-resistant subjects have increased Th17 cell counts compared to the AT of obese ones without signs of IR and neither to non-overweight ones. The study in adults with T2DM and nondiabetic ones revealed that blood from T2DM patients had shown increased circulating Th17 cells and elevated activation of Th17 signature genes (44). Furthermore, a positive correlation was found between BMI and percentage of Th17 cells in the obese T2DM cohort (44). However, there are only few papers evaluating this problem in children (37). In our study, in the group of all children, a statistically significant correlation was found between the frequency of Th17 lymphocytes and fasting insulin, HOMA-IR and QUICKI. Our study showed a statistically significant correlation between the frequency of Th17 cells and the concentration of glucose and insulin 2h after OGTT in overweight/obese children. These results support the observation, that pro-inflammatory Th17 cells have important contribution in glycemic homeostasis and the development of IR in children. Interestingly, we observed a trend towards an increase in QUICKI simultaneously with a statistically significant decrease in the frequency of Th17 cells after BMI reduction. It is worth noting that the change in the frequency of Th17 cells was observed in our study just after 3 months of the lifestyle changes and relatively small BMI SDS reduction.

In adults 5-10% losing of the initial body weight led to noticeably improve health by reducing obesity-related risk factors (45). This observation may suggest that inflammation and insulin resistance might be relatively easily reversible in children following lifestyle changes. This hypothesis however requires further investigation.

A marker routinely used in the assessment of inflammation is CRP- nonspecific acute-phase reactant that is synthesized in the liver. High-sensitivity CRP (hsCRP) - a more sensitive systemic inflammatory marker that indicates increased risk for metabolic complication of obesity is associated with other proinflammatory factors in plasma e.g., IL-6, Th1 and Th17 lymphocytes (46, 47). In our study, using standard CRP and not hsCRP, we did not find any difference between the plasma concentration in obese and overweight children and normal-weight ones, and no correlation between CRP and the frequency of Th17 cells. Th17 cell frequency assessment seems to be more sensitive than standard plasma CRP concentration, which suggests that it could be a valuable parameter for clinical use.

We realize that our study has some limitations. First of all- a relatively small group of patients who reduced the weight, which may have an impact on the statistical power of results. Secondly, our results show an association rather, than a direct cause-end-effect relationship, between Th17 cells and obesity related complications. However, this is one of the few studies assessing the immunological aspect of childhood obesity and their metabolic complications.

## Conclusions

Obesity in children is associated with increased percentage of peripheral Th17 cells. Weight reduction leads to significant decrease of circulating Th17 cells and improvement of lipid parameters. This significant reduction of proinflammatory Th17 cells is a promising finding suggesting that obesity-induced inflammation in children could be relatively easily reversible.

## Data Availability Statement

The original contributions presented in the study are included in the article/supplementary material. Further inquiries can be directed to the corresponding authors.

## Ethics Statement

The studies involving human participants were reviewed and approved by the Bioethics Committee at the Medical University of Warsaw (approval number : KB/52/A/2016; KB/61/2016). Written informed consent to participate in this study was provided by the participants’ legal guardian/next of kin.

## Author Contributions

DA-W conceptualized and designed the study, collected data, performed statistical analysis, prepared tables and figures, wrote and edited the manuscript. AK conceptualized and designed the study, interpreted the results, and revised the manuscript. AS-E designed the flow cytometry protocol, performed the flow cytometry analysis, prepared Figure 1 and reviewed the manuscript. AM performed anthropometric measurements, contributed to the writing of the "Anthropometry" part, and reviewed the manuscript. BP reviewed the manuscript. All authors contributed to the article and approved the version submitted.

## Conflict of Interest

The authors declare that the research was conducted in the absence of any commercial or financial relationships that could be construed as a potential conflict of interest.

## Publisher’s Note

All claims expressed in this article are solely those of the authors and do not necessarily represent those of their affiliated organizations, or those of the publisher, the editors and the reviewers. Any product that may be evaluated in this article, or claim that may be made by its manufacturer, is not guaranteed or endorsed by the publisher.
